# *Sorbaria sorbifolia* flavonoid derivative induces mitochondrial apoptosis in human hepatoma cells through Bclaf1

**DOI:** 10.3389/fphar.2024.1459520

**Published:** 2024-10-09

**Authors:** Jiaxin Chen, Haoyi Cheng, Chunhua Bai, Dandan Wang, Jinghao Fu, Jinge Hao, Yixuan Wang, Zhang Xuewu

**Affiliations:** College of Medicine, Yanbian University, Yanji, China

**Keywords:** *Sorbaria sorbifolia*, 4′,5-Dihydroxy-7-piperazinemethoxy-8-methoxy flavonoids, liver cancer, bcl-2-associated transcription factor 1, mitochondrial apoptosis

## Abstract

4′,5,7-Trihydroxy-8-methoxyflavone is an anticancer monomer component isolated from the traditional Chinese medicine *Sorbaria sorbifolia*. 4′,5-Dihydroxy-7-piperazinemethoxy-8-methoxy flavonoids (DMF) with good solubility and anti-tumor effects was obtained by chemical modification in the early stage. This study explored the mechanism by which DMF regulates the mitochondrial apoptosis of human hepatoma cells through Bcl-2-associated transcription factor 1 (Bclaf1). DMF inhibited the proliferation of human hepatoma cells in a concentration- and time-dependent manner and induced cell mitochondrial apoptosis. The molecular docking and cell assay results demonstrated that DMF inhibits Bclaf1 expression by binding to its active site. Lentivirus transfection was used to construct cells with stable knockout and overexpression of Bclaf1, and a Hep3B xenograft model was constructed in nude mice. The mechanism by which DMF induced the mitochondrial apoptosis of human hepatoma cells through Bclaf1 was further verified *in vitro* and *in vivo*. These findings indicated that DMF induced human hepatoma cell mitochondrial apoptosis through Bclaf1.

## 1 Introduction

Liver cancer is one of the most common malignant tumors globally. At present, interventional ablation, chemoradiotherapy, and biological immunotherapy are the main treatment methods, but they have severe side effects (Anwanwan et al., 2020; Sankar et al., 2020). Traditional Chinese medicine, used as an adjuvant therapy, offers advantages such as lower toxicity, reduced side effects, and more targets, which has attracted much attention from researchers in recent years.

*Sorbaria sorbifolia* is a Rosaceae plant, known for promoting blood circulation, reducing swelling, relieving pain, and treating the effects of traumatic injury. Its main components are flavonoids. In the early stage of our research, we isolated a compound from *S. sorbifolia* ethyl acetate extract and identified it as 5,2′,4′-trihydroxy-6,7,5′-trimethoxyflavone (TTF1). The compound was found to induce apoptosis in HepG2 cells through the mitochondrial pathway (Li et al., 2011), inhibit tumor angiogenesis (Liu et al., 2011), induce ERS-mediated apoptosis and inhibition of human hepatoma cells (Xiao et al., 2016b) in the form of the nanoparticles, inhibit angiogenesis, cell migration, and cell invasion in human hepatoma cells by regulating STAT3 (Xiao et al., 2016a), and induce protective autophagy during apoptosis by inhibiting the Akt/mTOR pathway and activating JNK in human hepatoma cells (Zhang et al., 2018). It exhibits a good anti-liver cancer effect. Our research group extracted and obtained 4′,5,7-trihydroxy-8-methoxyflavone from *S. sorbifolia*. Due to the poor solubility, after chemical modification, 4′,5-Dihydroxy-7-piperazinemethoxy-8-methoxy flavonoids (DMF) was screened as a compound with good anti-tumor effects. However, the anti-tumor mechanism remains unclear.

Bcl-2-associated transcription factor 1 (Bclaf1, also known as BTF) is a multifunctional protein located on human chromosome 6q23.3, and it encodes 17 transcriptional variants of different subtypes (Jiang et al., 2022). Bclaf1 is mainly localized in the nucleus, with a small distribution in the cytoplasm. The notable features of the Bclaf1 structure include its arginine–serine domain, bZIP domain, and MYB DNA-binding domain (Yu et al., 2022). It has been found that upregulated SMYD3 promoted bladder cancer progression by targeting Bclaf1 and activating autophagy (Shen et al., 2016). Bclaf1 binds to SPOP, thereby inhibiting PD-L1 ubiquitination and degradation and making cancer cells sensitive to checkpoint therapy, suggesting that Bclaf1 is a novel therapeutic target for enhancing anti-tumor immunity in HCC (Yu et al., 2024). Bclaf1 could bind to CircZFR, thereby preventing its ubiquitination and degradation to promote colorectal cancer cell proliferation and metastasis (Chen et al., 2024). Our previous studies found that Bclaf1 was the upstream regulator of HIF-1α in anoxic microenvironments. In addition, ginsenoside CK significantly inhibited the binding of Bclaf1 and HIF-1α, thereby suppressing HIF-1α-mediated glycolysis in anoxic human hepatoma cells and inhibiting their proliferation (Zhang et al., 2020). Curcumin induced mitochondrial apoptosis in human hepatoma cells by inhibiting Bclaf1 expression (Bai et al., 2022), which suggested that Bclaf1 is closely related to the development of hepatoma. To explore deeply the anti-tumor mechanisms of DMF through Bclaf1, this study explored the mitochondrial apoptosis effect by which DMF regulated Bclaf1 by *in vivo* and *in vitro* experiments, providing a theoretical and experimental basis for the development and utilization of *S. sorbifolia* flavonoid derivatives.

## 2 Materials and methods

### 2.1 Reagents

5-Fluorouracil (5-FU) was purchased from Yuanye Co., Ltd. (Shanghai, China). HPLC ≥ 98% (No. B25419), Dulbecco’s modified Eagle medium (DMEM) (No. 11965092), penicillin–streptomycin (No. 15140122), and fetal bovine serum (No. 302220F) were purchased from Gibco (Grand Island, NY, United States). The Cell Counting Kit-8 (No. 302220F) was purchased from APE×BIO (Houston, United States). The ECL chemiluminescence kit (No. WBK1S0100) was purchased from Millipore (Billerica, Massachusetts, United States). The ATP content kit (No. BC0305) and JC-1 mitochondrial membrane potential detection kit (No. C2006) were purchased from Solarbio (Beijing, China). Antibodies Bcl-2 (No. ab182858), Bax (No. ab32503), cytochrome C (Cyt-c) (No. ab133504), cleaved caspase-3 (No. ab32042), and Bclaf1 (No. ab181240) were purchased from Abcam (Cambridge, MA, United States). β-Actin (No. AC026) was purchased from ABclonal (Beijing, China). Horseradish-labeled goat anti-rabbit IgG (No. ZB-2306) was purchased from Zhongshan Jinqiao Co., Ltd. (Beijing, China). Horseradish-labeled goat anti-mouse IgG (No. 31430) was purchased from Thermo Co., Ltd. (Beijing, China).

### 2.2 Experimental animals

Thirty 5-week-old female BALB/C-NU mice, 17–19 g, were provided by Beijing Weitong Lihua Laboratory Animal Co., Ltd. (experimental animal production license: SCXK (Beijing) 2021-0006; certificate number: 110011221112896151; experimental animal use license: SYXK (Hubei) 2018-0101). The experiment program was approved by the Institutional Animal Care and Use Committee of Yanbian University (resolution number: 201501022).

### 2.3 Experimental cells

Human hepatoma cells HepG2 (No. FH0076), Hep3B (No. FH0861), and THLE-2 (No. FH1249) were purchased from Fuheng Biotechnology (Shanghai, China). QSG-7701(No. CL0264) was purchased from Fenghui Biotechnology Co., Ltd. (Changsha, Hunan, China).

### 2.4 Preparation of 4′,5,7-trihydroxy-8-methoxy flavone

First, commercial 2-methoxybenzene-1,3,5-triol (1.00 g, 5 mmol) was dissolved in CH_2_Cl_2_, anhydrous aluminum chloride was added at a catalytic amount, and chloroacetyl chloride was slowly dripped at room temperature (0.6 mL, 6 mmol) and refluxed for reaction at 40°C for 1 h. Thin-layer chromatography [unfolding agent: CH_2_Cl_2_ (v):MeOH (v) = 10:1] was used to monitor the reaction process. The reaction liquid dropped to room temperature, and a hydrochloric acid and ice water solution at a concentration of 1:1 was added to the mixture to begin the third extraction with ether. The organic layer was filtered and dried with the proper quantity of anhydrous sodium sulfate. The purification of 2-chloro-1-(2,4,6-trihydroxy-3-methoxyphenyl) thanone was obtained by column chromatography separation [eluent: v (CH_2_Cl_2_):v (petroleum ether) = 1:1].

Then, at room temperature, compound 2-chloro-1-(2,4,6-trihydroxy-3-methoxyphenyl) thanone reacted with p-hydroxybenzaldehyde (0.53 g, 5 mmol) and underwent an alkaline catalytic reaction in an ethanol solution for 24 h (pH = 11). Thin-layer chromatography was used to monitor the reaction process. After the reaction was completed, a 10% HCl solution was added to adjust the pH of the solution to neutral, and a bright yellow precipitate was observed, which was recrystallized with ethanol to obtain compound 4′,5,7-trihydroxy-8-methoxyflavone with a yield of 60%.

### 2.5 Preparation of 4′,5-dihydroxy-7-piperazinemethoxy-8-methoxy flavonoid

**FIGURE 1:**
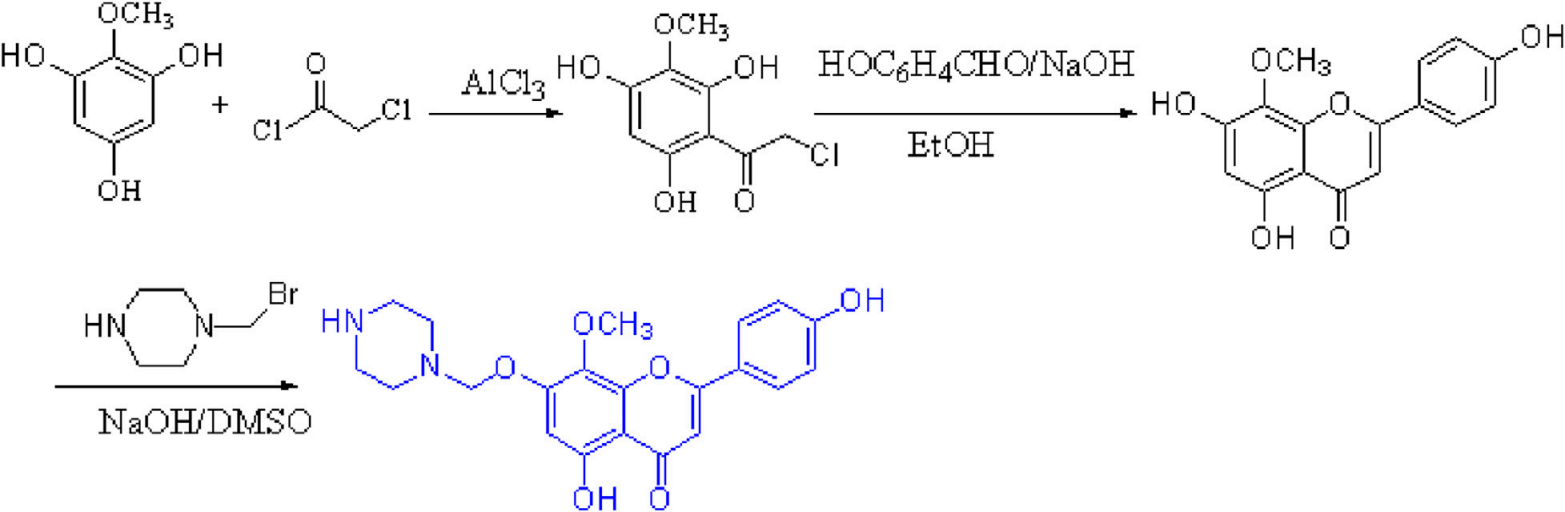
4′,5-Dihydroxy-7-piperazinemethoxy-8-methoxy flavonoids.

To a stirred solution of 4′,5,7-trihydroxy-8-methoxyflavone (1.0 g, 5 mmol) in dry dimethyl sulfoxide, a solution of 1-(bromomethyl) piperazine (0.6 g, 5 mmol) and NaOH (0.13 g, 5 mmol) was added at 0°C. The resulting mixture was stirred at room temperature for 5 h (reaction progress was monitored by TLC); after the reaction was completed, a 10% HCl solution was added to adjust the pH of the solution to neutral, resulting in the formation of a yellow precipitate, which was purified by recrystallization with EtOH to yield compound 4′,5-DMF. The yield was 71%. The structure of the target compound DMF was identified by IR, MS, 1H-NMR, and 13C-NMR (Figure 1).

### 2.6 Cell culture and proliferation experiment

HepG2, Hep3B, and THLE-2 cells were cultured in a culture medium (DMEM:fetal bovine serum:penicillin–streptomycin = 100:1:1). QSG-7701 cells were cultured in a culture medium (RPMI:fetal bovine serum:penicillin–streptomycin = 100:1:1) in a humid incubator (37°C, 5% CO_2_). When the cells reached the logarithmic growth phase, CCK-8 was used to detect cell proliferation ability, and 100 μL of cell suspension was added to the pores of the 96-well plate so that the cell density in each hole was 5 × 10^3^ cells/mL. After the cells were attached to the wall, the HepG2 and Hep3B cells were divided into the following groups: control group, DMF treatment group (25, 50, 100, 200, and 250 μM), sgRNA group, and sgRNA + DMF group. The THLE-2 and QSG-7701 cells were divided into the following groups: control group and DMF treatment group (50, 100, and 200 μM). Each group was set up with five compound pores, and 10 μL CCK-8 solution was added to each group and incubated for 1 h. OD values of each pore solution were detected at a wavelength of 490 nm using an enzyme-labeled instrument, and the data were recorded to calculate the cell growth inhibition rate.

Cell growth inhibition rate (%) = 1−[(experimental group OD value−blank group OD value)/(negative control group OD value−blank group OD value)] × 100%.

Cell viability (%) = (experimental group OD value−blank group OD value)/(negative control group OD value−blank group OD value)] × 100%.

### 2.7 Determination of the ATP content

The cells of the logarithmic growth stage were divided into DMF treatment groups (0, 50, 100, and 200 μM) and a positive control group (5-FU). After administering the drug, 5 × 10^6^ cells were taken from each group; 1 mL of extraction solution was added, crushed using ultrasonic waves, and then centrifuged for 10 min. The supernatant was obtained, and chloroform was added to the mix. The supernatant was obtained after centrifugation.

According to the instructions of the ATP test kit, the corresponding reagent was added to each tube in turn and then fully mixed. The absorbance value A1 at 10 s was determined at 340 nm, and then the sample was placed in a 37-C water bath for 3 min. The absorbance value was immediately measured and recorded as A2. The ATP content of the sample was tested according to the manufacturer’s instructions.

### 2.8 CRISPR/Cas9-mediated *Bclaf1* gene knockout experiment

HepG2 and Hep3B cells were divided into a blank control group and an sgRNA group. The next day, 1 mL of high-sugar medium was replaced, and 20 μL of lentivirus vector was added to the sgRNA group. The complete culture medium was replaced after incubation for 12 h. After incubation for 48 h, 6 μg/mL of mycillin was added to each well for screening, and stable cell lines with the *Bclaf1* gene knocked out were obtained (Chen et al., 2022).

### 2.9 Western blotting

The cell or tumor tissue proteins were extracted, and then, the protein concentration was determined using the BCA method. The loading volume was calculated according to the sample concentration. After separation by sodium dodecyl sulfate polyacrylamide gel electrophoresis (at 100 V for 120 min), the corresponding polyvinylidene fluoride (PVDF) membrane was clipped according to the molecular weight of the transferred target protein. The proteins were transferred to the PVDF membrane (100 V, 30–90 min). The membrane was then blocked with 5% skim milk. The primary antibody was diluted with TBST: Bcl-2 (1:2,500), Bax (1:2,500), Cyt-c (1:2,000), cleaved caspase-3 (1:2,000), Bclaf1 (1:2,500), and β-actin (1:30,000) and was incubated overnight at 4°C. On day 2, the anti-rabbit secondary antibody (1:5,000) was incubated for 2 h, and ECL chemiluminescence solution was added. The protein was detected using a bioanalytical imaging system (Cycloud, Beijing, China).

### 2.10 Annexin V/FITC double staining

HepG2 and Hep3B cells were inoculated in 6-well plates with 1 × 10^6^ cells per well and divided into an unstained group, Annexin V group, PI group, DMF (0, 50, 100, and 200 µM) administration group, 5-FU (10 µM) group, sgRNA group, sgRNA + DMF group, Bclaf1-overexpression group, and Bclaf1-overexpression + DMF group. They were digested with a pancreatic enzyme without EDTA, and pre-cooled PBS was added to prepare the cell suspension and centrifuged at 3,000 g for 2 min. Then, 1 mL PBS was added to the cell mass. Centrifugation was performed at 3,000 g for 2 min, after which PBS was discarded. Next, 100 μL 1 × binding buffer was added, and 10 μL was removed from each tube. Subsequently, 5 µL Annexin V was added and incubated for 20 min, followed by the addition of 5 µL PI, which was incubated in the dark for 5 min. Finally, 400 μL 1 × binding buffer was added to each tube, and the apoptosis rate of cells was detected by flow cytometry (Beckman Coulter Corporation, United States).

### 2.11 JC-1 fluorescent probe method

HepG2 and Hep3B cells were divided into a negative control group, DMF (100 µM) administration group, 5-FU (10 µM) administration group, sgRNA group, and sgRNA + DMF group, Bclaf1-overexpression group, Bclaf1-overexpression + DMF group, and carbonyl cyanide 3-chlorophenylhydrazone (CCCP) group. After incubating for 48 h, the cells in the CCCP group were treated with the JC-1 staining solution for 40 min. The culture medium was discarded, and the JC-1 dyeing solution was added to each well and incubated for 60 min. The JC-1 buffer was diluted with distilled water (1:4), washed with the diluted JC-1 buffer, and then photographed under a 400× fluorescence microscope (Olympus, Japan) (Chen et al., 2022).

### 2.12 Immunofluorescence

HepG2 and Hep3B cells were inoculated in the 6-well plate; the next day, the blank control group, DMF (0, 50, 100, and 200 µM) group, and 5-FU (10 µM) group were treated with drugs and culture medium and incubated for 48 h. The culture medium was discarded, and 4% paraformaldehyde was incubated for 10 min. After infiltration of 0.2% Triton X-100 for 20 min, an appropriate amount of 5% BSA was added to each well and closed for 1 h. Then, 100 µL Bclaf1 (1:200) was uniformly dripped onto the tablet, which was then placed in a wet box sheltered from light and refrigerated at 4°C overnight. The next day, 200 µL fluorescein (1:100) was added away from light and incubated at 37°C for 1 h. Then, DAPI was cleaned with PBS, stained for 5 min under a 400× fluorescence microscope, and photographed.

### 2.13 Construction of a stable transmutation strain with the overexpression of Bclaf1

HepG2 and Hep3B cells were inoculated in 6-well plates. The next day, the viral stock solution was melted and diluted with fresh medium containing 8 μg/mL polybrene according to the appropriate MOI value. The lentivirus diluent was added to the cells. The next day, 2 μg/mL puromycin was added to the cells and cultured in an incubator for 24 h. Cells with stable overexpression of Bclaf1 were screened, and the infection effect was verified by Western blotting.

### 2.14 Establishment of a nude mouse transplanted tumor model

In nude mice, 1 × 10^7^ Hep3B cells were inoculated in the right armpit (forelimb), and when the tumor grew to an average volume of 80–100 mm^3^, the drug was administered (intravenous injection). Before administration, the nude mice were weighed, and the tumor volume was measured. The nude mice were divided into the lysozyme control group, DMF (5, 10, and 20 mg/kg) group, and positive drug 5-FU (15 mg/kg) group. In the experiment, if the tumor volume of a single mouse exceeded 2,000 mm^3^, the experiment was terminated. When the mean tumor volume of mice in the control group exceeded 1,000 mm^3^ or when the tumor size of the control group and the experimental group mice was different, all nude mice were euthanized. The anesthetic was 20% urethane, intraperitoneally injected at a dose of 200 μL/20 g. The weight and tumor volume of nude mice were measured, and the tumors were collected.

Tumor volume calculation formula: tumor volume (mm^3^) = 1/2 × (tumor long diameter × tumor short diameter^2^).

### 2.15 Immunohistochemical experiments

Tissue wax blocks were prepared by transplanting tumors in nude mice. After the sections were soaked in xylene for 10 min and dehydrated in a gradient, the antigen repair solution was added. The sections were kept in a slightly boiling state for 30 min. Two drops of 3% hydrogen peroxidation–methanol solution were added to the sections for 10 min, and 200 μL 5% BSA was then added to the sections for 20 min. Each slice was added with 100 μL of primary antibody Bclaf1 (1:200), incubated at 37°C in a wet box for 2 h, then dropped using an 80-μL enhancer, and placed for 30 min. Then, 100 μL of secondary antibody was added and incubated at 37°C for 30 min. Then, 50 μL of the DAB solution and hematoxylin dye solution were added to the tissue sections for 10 min, dehydrated with an ethanol gradient, and transplanted with xylene for 20 min. Then, neutral gum was added to seal the tablets.

### 2.16 Molecular docking experiments of target compounds

Molecular simulation and docking experiments were carried out using the PyRx docking program. The protein crystal structure of Bclaf1 was obtained from the Protein Crystal Database (PDB ID: 7RJR) with a resolution of 1.45 Å. The initial structure of the Bclaf1 protein crystal was treated with default parameters. Here, 1, 2-ethylene glycol was used as the ligand center, and the optimized protein crystal structure and compound were simulated. The score (−5.4290) of the result was treated using PyMOL to evaluate the effect of the compound at the molecular level.

### 2.17 Statistical analysis

Data analysis was performed using GraphPad Prism 6. The data were expressed as the mean ± standard deviation (SD). Differences between groups were analyzed by one-way analysis of variance and Student’s t-test. *p* < 0.05 was considered statistically significant, and *p* < 0.01 was considered a highly significant difference.

## 3 Results

### 3.1 DMF inhibited human hepatoma cell proliferation

**FIGURE 2:**
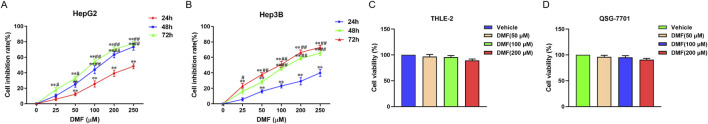
Effects of DMF on the proliferation of human hepatoma cells and normal hepatocytes. **(A)** HepG2; **(B)** Hep3B; **(C)** THLE-2; **(D)** QSG-7701, data were mean ± SD, n = 3; the results were compared with vehicle, **P < 0.01, the results were compared with 24h groups, ^#^ *P* < 0.05, ^##^ *P* < 0.01.

The effects of DMF on the proliferation of HepG2 and Hep3B cells were detected using the CCK-8 assay. The cells were treated with DMF (25, 50, 100, 200, or 250 μM) for 24, 48, or 72 h. The results indicated that DMF inhibited the proliferation of human hepatoma cells in a time- and concentration-dependent manner. The IC_50_ values of DMF after treatment for 48 h in HepG2 and Hep3B cells were 121.7 and 130.4 μM, respectively (Figures 2A, B). The DMF concentration gradient selected for subsequent experiments was 50, 100, and 200 μM. The toxicity of DMF to normal hepatocytes was further examined, and the results showed that there was no significant difference in cell viability between TELE-2 and QSG-7701 normal hepatocytes after DMF treatment (Figures 2C, D).

### 3.2 DMF induced mitochondrial apoptosis in human hepatoma cells

**FIGURE 3:**
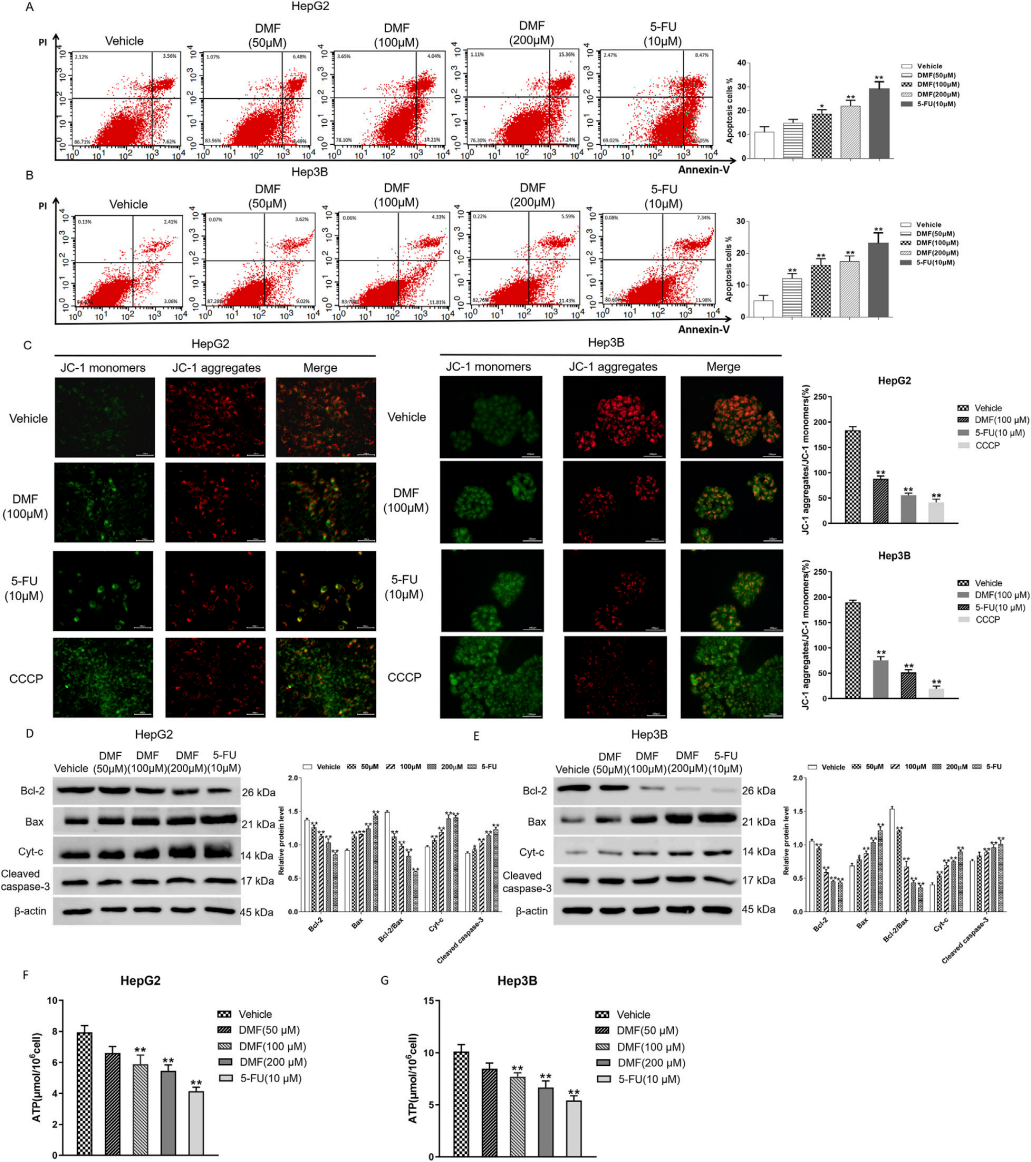
DMF induced mitochondrial apoptosis in human hepatoma cells. **(A,B)** Flow cytometry was used to detect the effect of DMF on apoptosis rates. **(C)** The JC-1 fluorescent probe method was used to detect changes in the mitochondrial membrane potential (400×). **(D,E)** Western blotting detected the expression levels of proteins, and the results were statistically analyzed. **(F,G)** Content of ATP in the cells; all the results were compared with those of the vehicle. *p < 0.05 and **p < 0.01.

Flow cytometry was used to detect the change in the apoptosis rate in cells treated with DMF for 48 h. The experimental results indicated that the percentage of apoptotic cells increased in a DMF concentration-dependent manner. The percentages of apoptotic HepG2 cells after treatment with 50, 100, and 200 μM DMF and positive drug 5-FU were 11.06%, 14.85%, 18.66%, 21.96%, and 29.08%, respectively, and the percentages of apoptotic Hep3B cells after these treatments were 5.11%, 12.04%, 16.24%, 17.45%, and 23.10%, respectively (Figures 3A, B). HepG2 and Hep3B cells were examined using the JC-1 fluorescence probe method after DMF treatment. Following treatment, the red fluorescence was weakened, whereas the green fluorescence was enhanced, indicating decreased mitochondrial membrane potential and increased cell permeability (Figure 3C). To further explore the effects of DMF on mitochondrial apoptosis in human hepatoma cells, Western blotting was performed to detect the expression of Bcl-2, Bax, Cyt-c, and cleaved caspase-3 after DMF treatment. The results illustrated that the Bcl-2/Bax ratio was significantly decreased after DMF treatment compared with the findings in the negative control group (*P* < 0.01), whereas Cyt-c and cleaved caspase-3 expression was increased. Similar results were obtained after 5-FU treatment (Figures 3D, E). ATP is the main energy molecule in the cells, and its content can reflect the energy production capacity of mitochondria. The experimental results showed that compared with the control group, the content of ATP in the cells decreased significantly after DMF treatment (Figures 3F, G), indicating that DMF destroyed the integrity of the mitochondria and affected the function.

### 3.3 DMF inhibited Bclaf1 expression in human hepatoma cells

**FIGURE 4:**
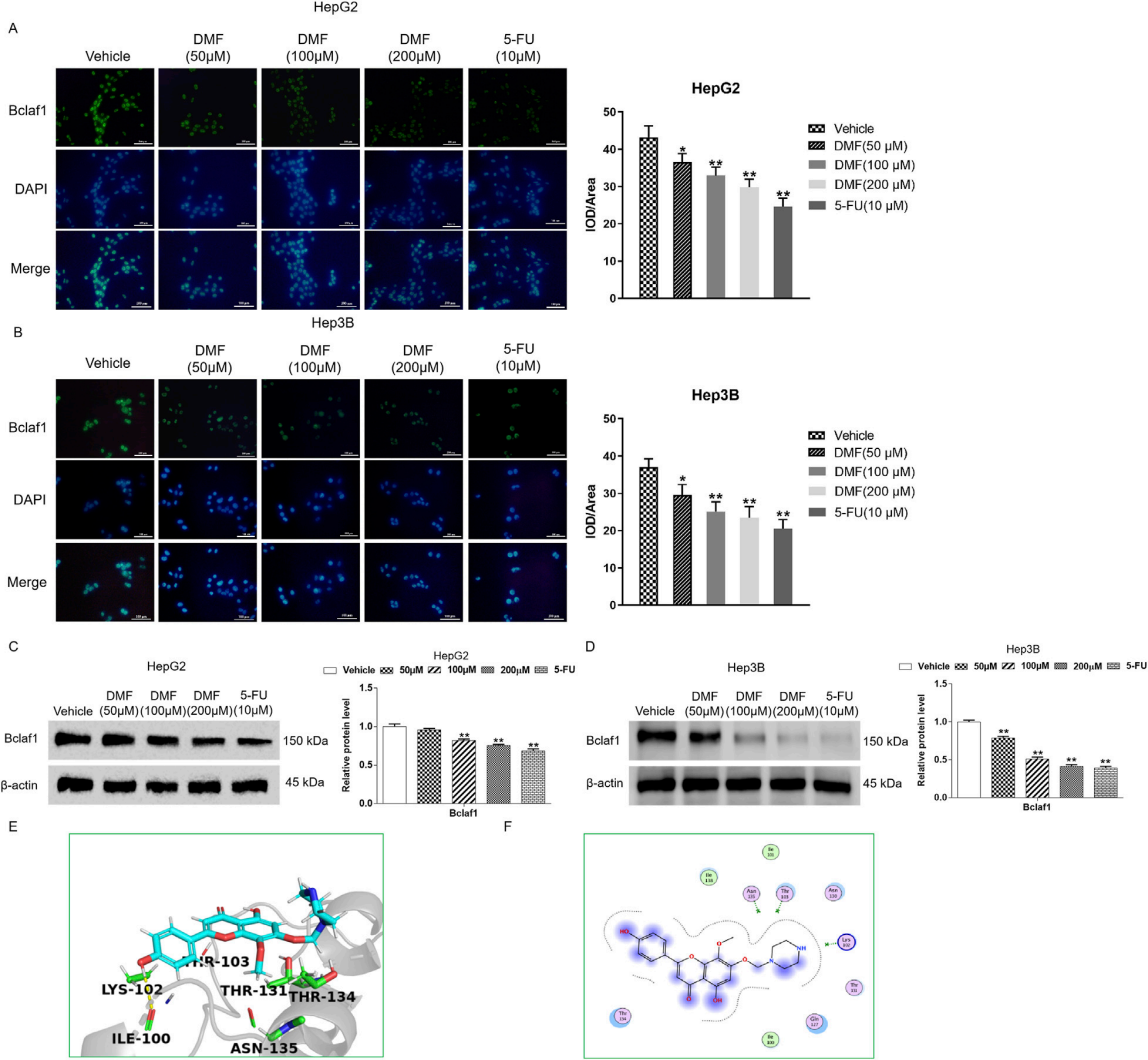
DMF inhibited Bclaf1 expression in human hepatoma cells. **(A,B)** Immunofluorescence was used to detect the expression of Bclaf1 after the cells were treated with DMF (400×). **(C,D)** Western blotting was used to detect the expression level of Bclaf1, and the results were statistically analyzed. All the results are compared with those of the vehicle. *p < 0.05 and **p < 0.01. **(E,F)** Docking result of DMF and Bclaf1 (PDB ID: 7RJR).

Immunofluorescence detection revealed that Bclaf1 was expressed in both the nucleus and cytoplasm of untreated human hepatoma cells. After DMF treatment, the fluorescence intensity of Bclaf1 decreased in a concentration-dependent manner (Figures 4A, B). Western blotting illustrated that DMF significantly decreased Bclaf1 expression versus the control (*p* < 0.01; Figures 4C, D). The molecular docking of DMF in Bclaf1 was studied *in vitro*. The results demonstrated the optimal binding location and interaction between the receptor and target (Figures 4E, F). The 4′- and 5-hydroxyl groups of DMF formed hydrogen bonds with ILE-100 and THR-134 in the active center of Bclaf1, respectively, and the oxygen atoms at positions 1 and 7 of DMF formed hydrogen bonds with the amino acid residues THR-103 and THR-131 of Bclaf1, respectively. The -NH moiety of the DMF piperazine ring formed a hydrogen bond with LYS-102 of Bclaf1.

### 3.4 DMF induced mitochondrial apoptosis in human hepatoma cells with stable Bclaf1 knockout

**FIGURE 5:**
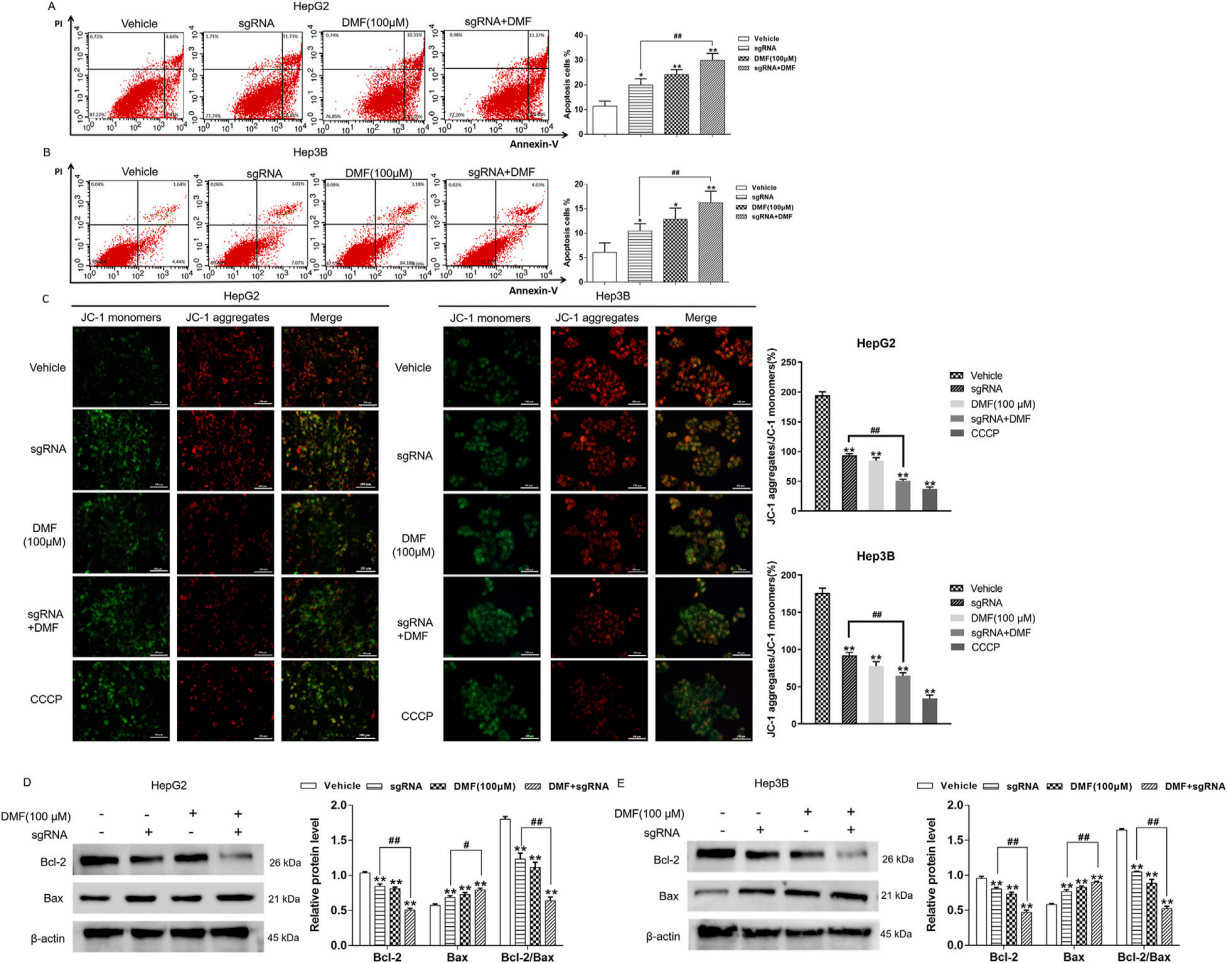
DMF induced mitochondrial apoptosis in human hepatoma cells with stable Bclaf1 knockout. **(A,B)** Flow cytometry was used to detect the changes in apoptosis rates. **(C)** The JC-1 fluorescence probe was used to detect the effect of DMF on the mitochondrial membrane potential (400×). **(D,E)** Western blotting was used to detect the expression of mitochondrial apoptosis-related proteins, Bcl-2 and Bax. Statistical analysis was performed; the results were compared with those of the vehicle. *p < 0.05 and **p < 0.01. The sgRNA group was compared with the sgRNA + DMF group. ^#^ *p* < 0.05 and ^##^ *p* < 0.01.

Annexin V/FITC double-staining revealed that the apoptosis rate of human hepatoma cells was increased after Bclaf1 knockout compared with the vehicle (*p* < 0.05). The apoptosis rate of HepG2 cells was further increased by DMF treatment, and rates in the groups were 11.37%, 19.96%, 24.19%, and 29.91%, respectively. The apoptosis rates of Hep3B cells in all groups were 6.10%, 10.47%, 12.85%, and 16.24%, respectively (Figures 5A, B). To further explore the role of DMF in inducing mitochondrial apoptosis in human hepatoma cells through Bclaf1, the fluorescence probe method was applied. Bclaf1 knockout resulted in increased green fluorescence, decreased red fluorescence, and decreased mitochondrial membrane potential in cells, indicating that Bclaf1 knockout induced mitochondrial apoptosis in human hepatoma cells. After DMF treatment in cells with stable Bclaf1 knockout, the mitochondrial membrane potential was further decreased (Figure 5C). After Bclaf1 knockout, Bcl-2 expression was downregulated, Bax expression was increased, and the Bcl-2/Bax ratio was decreased versus the control findings (*p* < 0.01), indicating that mitochondrial apoptosis was induced in human hepatoma cells after Bclaf1 knockout (Figures 5D, E).

### 3.5 Bclaf1 overexpression inhibited mitochondrial apoptosis in human hepatoma cells

**FIGURE 6:**
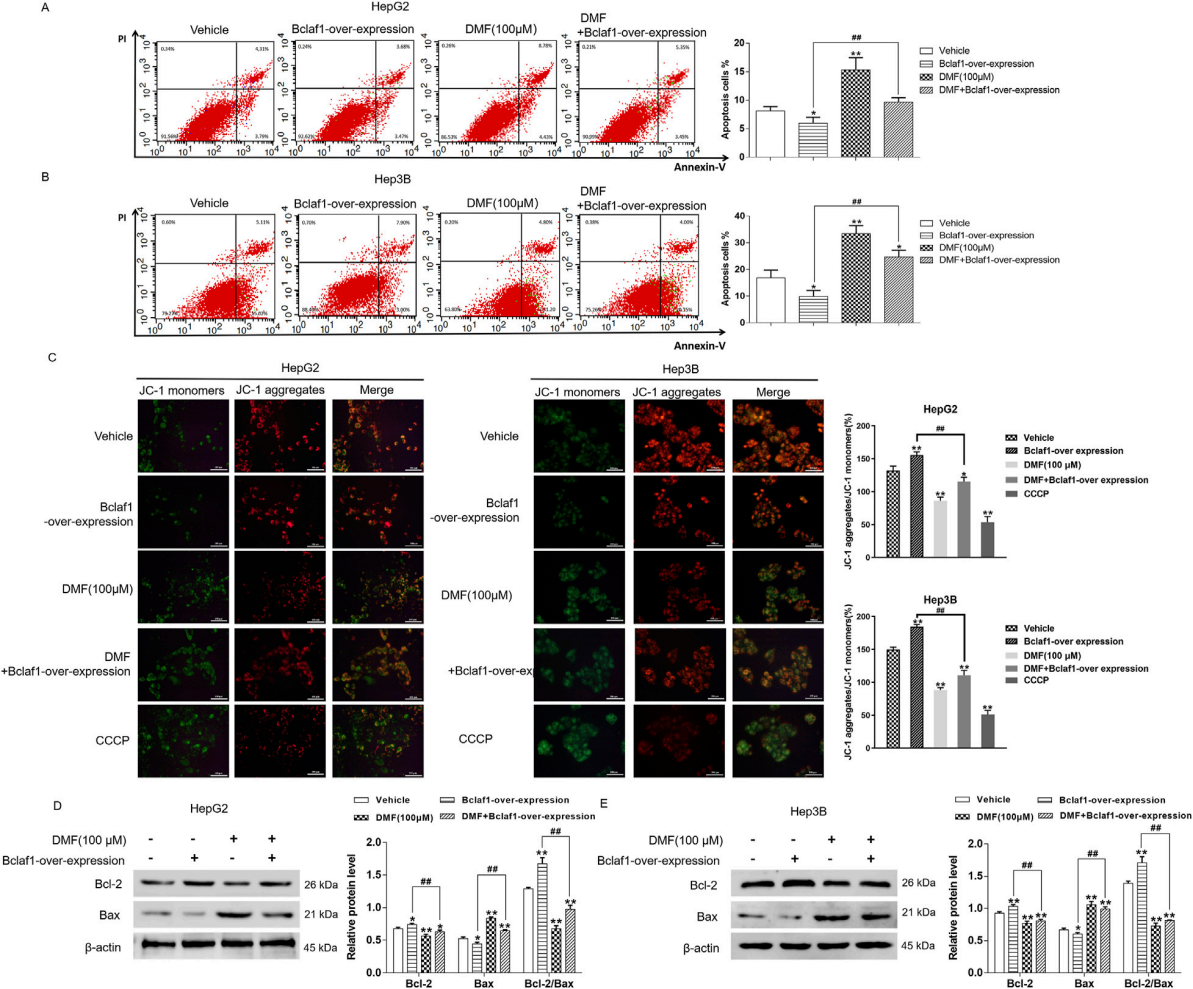
Overexpression of Bclaf1 inhibited mitochondrial apoptosis in human hepatoma cells. **(A,B)** Flow cytometry was used to detect the changes in apoptosis rates. Statistical analysis was performed. **(C)** The JC-1 fluorescence probe method was used to detect the effect of DMF on the mitochondrial membrane potential (400×). **(D,E)** Western blotting was used to detect the expression of mitochondrial apoptosis-related proteins, Bcl-2 and Bax. Statistical analysis was performed. The results were compared with those of the vehicle. *p < 0.05 and **p < 0.01. The Bclaf1-overexpression group was compared with the DMF + Bclaf1-overexpression group. ^##^ *p* < 0.01.

Flow cytometry revealed that the apoptosis rate of human hepatoma cells was decreased by Bclaf1 overexpression (*P* < 0.05). DMF treatment significantly increased the apoptosis rate (*p* < 0.01) in HepG2 cells, and the apoptosis rates in all groups were 8.13%, 5.98%, 15.32%, and 9.67%, respectively. The apoptosis rates in the groups in Hep3B cells were 16.87%, 9.89%, 33.46%, and 24.71%, respectively (Figures 6A, B). In the JC-1 fluorescence probe assay, Bclaf1 overexpression resulted in decreased green fluorescence and increased red fluorescence, indicating that the mitochondrial membrane potential of human hepatoma cells was increased. Compared with the results in the blank control group, the green fluorescence was enhanced, the red fluorescence was weakened, and the mitochondrial membrane potential of the cells was decreased by DMF treatment (Figure 6C). After Bclaf1 overexpression was confirmed by Western blotting, the expression of Bcl-2, a key protein of mitochondrial apoptosis in hepatoma cells, was increased and that of Bax was decreased, and the Bcl-2/Bax ratio was increased compared with the findings in the blank control group (all *p* < 0.01). After DMF treatment, the Bcl-2/Bax ratio was decreased, indicating that mitochondrial apoptosis occurred in human hepatoma cells (Figures 6D, E).

### 3.6 DMF inhibited the growth of transplanted tumors and induced the mitochondrial apoptosis of the transplanted tumor in nude mice

**FIGURE 7:**
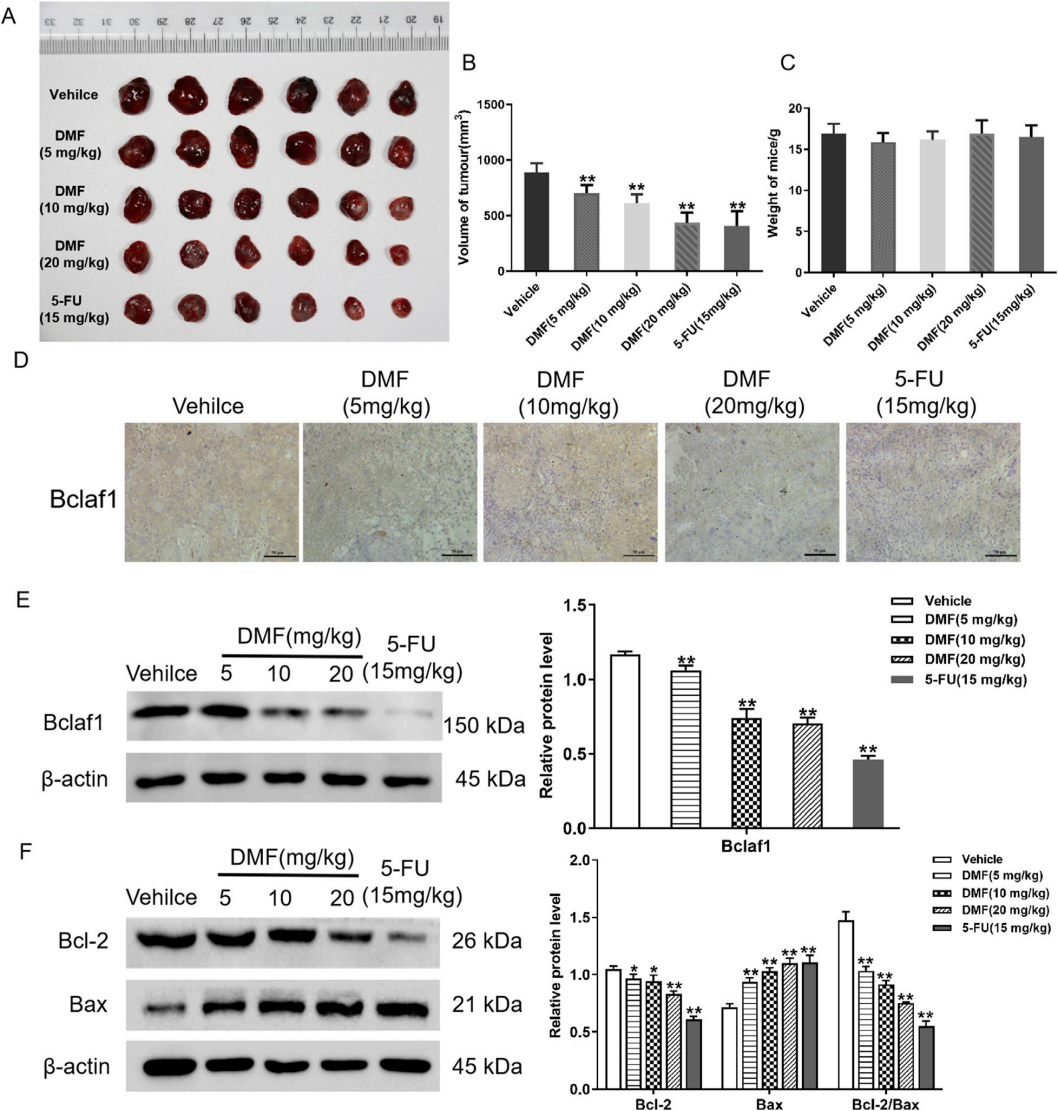
DMF inhibited the growth of transplanted tumors in nude mice. **(A)** Photograph of transplanted tumors taken from nude mice. The concentrations of DMF used to treat nude mice are 5 mg/kg, 10 mg/kg, and 20 mg/kg. The concentration of the positive control, 5-FU, was 15 mg/kg. **(B)** Volume of the transplanted tumor in nude mice (mm^3^). **(C)** Changes in body weight after nude mice were treated with DMF (5, 10, and 20 mg/kg) or 5-FU (15 mg/kg). **(D)** Immunohistochemical of Bclaf1 expression in tumor tissue (200×). **(E)** Western blot analysis of Bclaf1 expression in tumor tissue. **(F)** Western blot detection of the expression of Bcl-2 and Bax in tumor tissues; the results were compared with those of the vehicle. *p < 0.05 and **p < 0.01.

To further verify the anti-tumor effects of DMF, the inhibitory effects of DMF (5, 10, and 20 mg/kg) and the positive control 5-FU (15 mg/kg) on the transplanted tumor were detected by establishing a nude mouse transplanted tumor model. The results illustrated that DMF significantly inhibited tumor growth (*p* < 0.01; Figures 7A, B), but there was no significant change in the body weight in each group (Figure 7C). Immunohistochemistry illustrated that compared with the vehicle, Bclaf1 expression was decreased by DMF treatment in a dose-dependent manner. Using extracted histones, Western blotting indicated that DMF inhibited the expression of Bclaf1 (Figures 7D, E). The tumor tissue protein was extracted, and Western blotting indicated that DMF treatment resulted in decreased Bcl-2 expression and increased Bax expression in tumor tissues, resulting in a lower Bcl-2/Bax ratio (all *p* < 0.01). The Bcl-2/Bax ratio was the smallest in the positive control group (Figure 7F).

## 4 Discussion

Liver cancer is the sixth most common cancer worldwide and the second leading cause of cancer-related death (Li et al., 2022). Traditional chemotherapy has strong side effects, and it is associated with low survival rates. Chinese medicine molecular targeted therapy has fewer overall side effects, making it an important adjuvant anti-tumor therapy for chemotherapy, radiotherapy, and surgery (Hao et al., 2021; Rodríguez-Landa et al., 2022). 4′,5,7-Trihydroxy-8-methoxyflavone was extracted from the traditional Chinese medicine *S. sorbifolia*, and after chemical modification, DMF was selected for the assessments of anti-tumor effects because of its better solubility. Following DMF treatment for 48 h, the growth inhibition rate of human hepatoma cells was significantly increased, and there was no significant difference in the activity of normal hepatocytes. In this study, HepG2 and Hep3B cells were targeted, and Annexin V/PI double-staining revealed that the apoptosis rate increased significantly after 100 μM DMF treatment compared with that in the blank control group, demonstrating that DMF can induce apoptosis of human hepatoma cells. Apoptosis can be categorized into the death receptor-mediated extrinsic pathway, mitochondrial pathway-mediated apoptosis, granzyme B-mediated apoptosis, and endoplasmic reticulum stress-induced apoptosis (Wang et al., 2018; Cheng and Ferrell, 2018). Further exploration of the apoptosis pathway induced by DMF revealed that DMF treatment decreased the mitochondrial membrane potential, as detected the by JC-1 probe. When cells are subjected to internal hypoxia, injury, and other damages, Bcl-2 family proteins are key regulatory factors activated in the mitochondrial apoptosis pathway, including the pro-apoptotic proteins Bax and Bak and the anti-apoptotic protein Bcl-2 (Schofield and Schafer, 2023; Bhosale et al., 2023). Bax reduces the membrane potential, changes the membrane permeability, induces Cyt-c release in the cytoplasm, and forms an apoptosis complex with apoptotic protease-activating factor-1. Then, by recruiting and activating pro-caspase-9, the holoenzyme caspase 9 is formed, and caspases 3 and 7 are further activated as effectors. Consequently, the caspase cascade is initiated, and more than 100 substrates, such as actin, are cleaved, leading to apoptosis (Chu et al., 2021; Kashyap et al., 2021). Western blotting demonstrated that DMF decreased the Bcl-2/Bax ratio versus the control findings (*p* < 0.01). A decreased Bcl-2/Bax ratio is an important feature of mitochondrial apoptosis. Experimental detection demonstrated that Cyt-c and cleaved caspase-3 expression was increased. When mitochondrial apoptosis occurred in cells, the function of mitochondria was affected. A microanalysis found that after DMF treatment, the content of ATP in cells was significantly decreased, indicating that the integrity of mitochondria was destroyed. The above experimental results showed that after DMF treatment, the expression level of pro-apoptotic factor Bax increased, the expression level of anti-apoptotic factor Bcl-2 decreased, and the value of Bcl-2/Bax decreased, which decreased the mitochondrial membrane potential, opened the mitochondrial membrane permeability transition hole, promoted the release of pro-apoptotic proteins such as Cyt-c and cleaved caspase-3, and finally caused apoptosis.

Bclaf1, which is rich in arginine–serine-binding domains and contains MYB DNA-binding and bZIP domains, is involved in a variety of biological behaviors (Mou et al., 2020; Wen et al., 2019; Shao et al., 2016). This study revealed that Bclaf1 is highly expressed in human hepatoma cells and mainly distributed in the nucleus through immunofluorescence detection. DMF decreased Bclaf1 expression in a concentration-dependent manner. Molecular docking analysis showed that the 4′- and 5-hydroxyl groups of DMF formed hydrogen bonds with ILE-100 and THR-134, respectively, in the active center of Bclaf1, and the oxygen atoms at positions 1 and 7 of DMF formed hydrogen bonds with the amino acid residues THR-103 and THR-131 of Bclaf1, respectively. The -NH moiety of the DMF piperazine ring formed a hydrogen bond with LYS-102 of Bclaf1, which suggested that DMF could bind to the active site of Bclaf1 to inhibit its expression. Further probing the mechanism by which DMF induces mitochondrial apoptosis of human hepatoma cells through Bclaf1 showed that Bclaf1 knockout led to an increased apoptosis rate in human hepatoma cells by flow cytometry, and after the action and stability of DMF, the apoptosis rate was further increased. Bclaf1 knockout resulted in decreased mitochondrial membrane potential, which was further decreased in the DMF + sgRNA group, in addition to a decreased Bcl-2/Bax ratio. These results indicated that DMF could induce mitochondrial apoptosis in hepatoma cells through Bclaf1. To further verify this effect, after Bclaf1 overexpression, relevant indicators were detected. The results illustrated that Bclaf1 overexpression depressed mitochondrial apoptosis in human hepatoma cells and that DMF induced mitochondrial apoptosis in cells by inhibiting Bclaf1.

*In vitro* experiments demonstrated that DMF induced the mitochondrial apoptosis of human hepatoma cells through Bclaf1, thus inhibiting their proliferation. To verify the anti-tumor effects of DMF, a transplanted tumor model was constructed in nude mice, and the mice were euthanized after treatment with 5, 10, or 20 mg/kg DMF or 15 mg/kg 5-FU. The transplanted tumor was excised, and the tumor volume was calculated. DMF significantly inhibited tumor growth compared with the blank control group findings (*p* < 0.01), and the body weight of nude mice did not change significantly. To explore the mechanism by which DMF induced mitochondrial apoptosis through Bclaf1, immunohistochemical tests revealed that DMF treatment decreased Bclaf1 expression. Then, histones were extracted to detect Bcl-2 and Bax, and the results indicated that after DMF treatment, the Bcl-2/Bax ratio was decreased, indicating that DMF can induce mitochondrial apoptosis in liver cancer.

In summary, 4′,5,7-trihydroxy-8-methoxyflavone was extracted from *S. sorbifolia,* a traditional Chinese medicine, and structural modification resulted in the identification of DMF, which inhibited human hepatoma cell proliferation and induced mitochondrial apoptosis. The effect of DMF was linked to its targeting of the multi-functional protein Bclaf1. Inhibition of Bclaf1 expression can induce mitochondrial apoptosis in human hepatoma cells, and the anti-tumor effect of DMF was further verified through the construction of a nude mouse transplanted tumor model. Therefore, our study is expected to provide an experimental basis for DMF as an effective *S. sorbifolia* flavonoid derivative to treat liver cancer with Bclaf1 as its target and provide new ideas for the targeted therapy of liver cancer in clinical practice.

## 5 Conclusion

DMF induces mitochondrial apoptosis in human hepatoma cells through Bclaf1.

## Data Availability

The original contributions presented in the study are included in the article/Supplementary Material; further inquiries can be directed to the corresponding author.
