# Supplementary material for: Sorbaria sorbifolia flavonoid derivative induces mitochondrial apoptosis in human hepatoma cells through Bclaf1
Source: Front Pharmacol. 2024 Oct 9;15:1459520. doi: 10.3389/fphar.2024.1459520 (PMC11496133; doi:10.3389/fphar.2024.1459520)

Supplementary Material

Figure 3E

Bcl-2

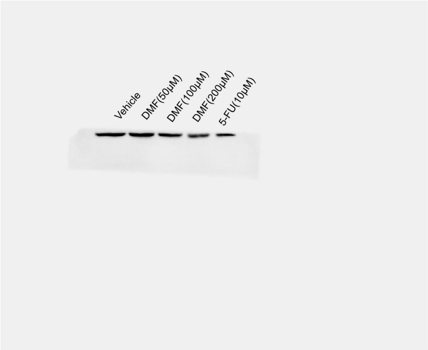

Bax

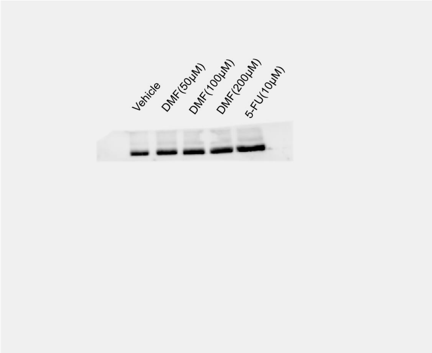

Cyt-c

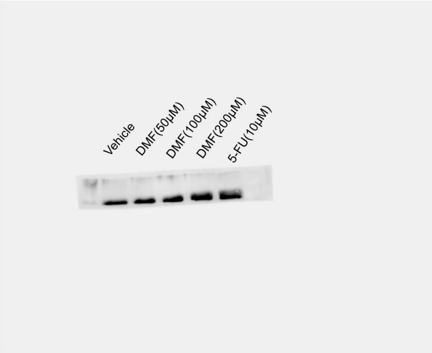

Cleaved caspase-3

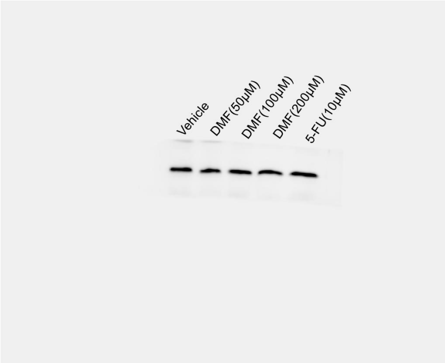

β-actin

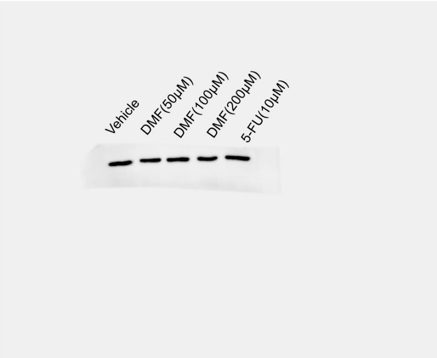

Figure 3F

Bcl-2

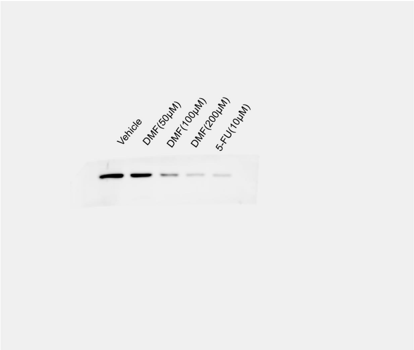

Bax

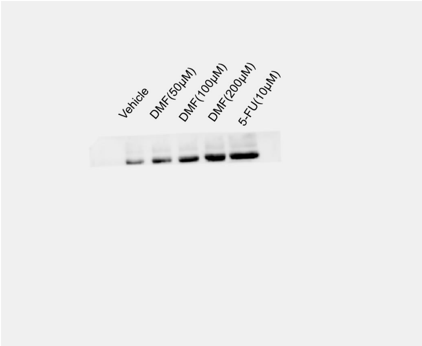

Cyt-c

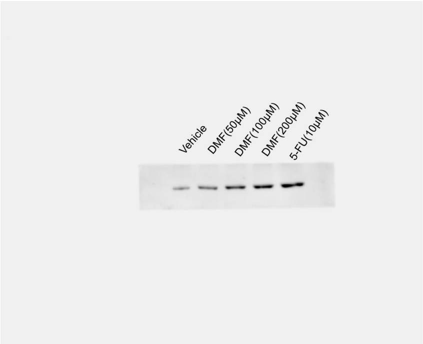

Cleaved caspase-3

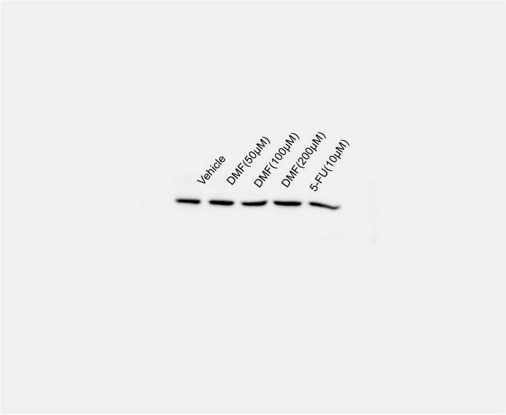

β-actin

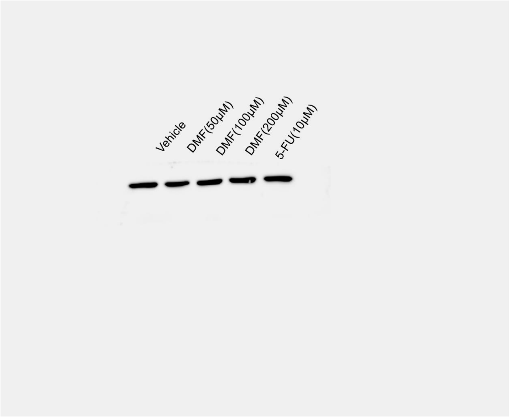

Figure 4C

Bclaf1

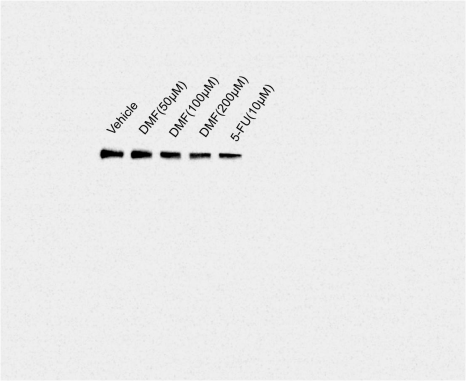

β-actin

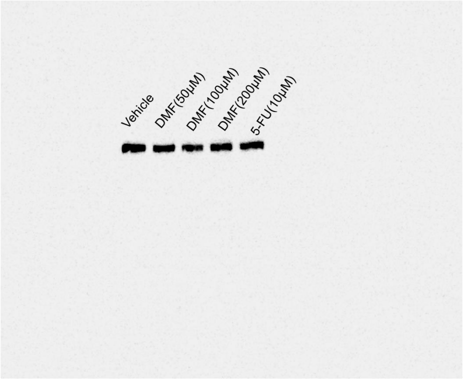

Figure 4D

Bclaf1

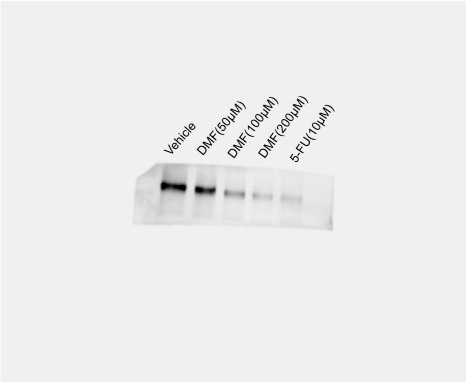

β-actin

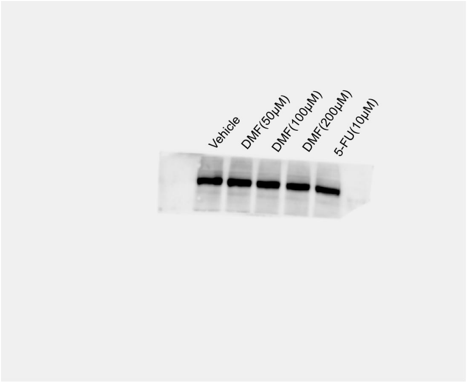

Figure 5E

Bcl-2

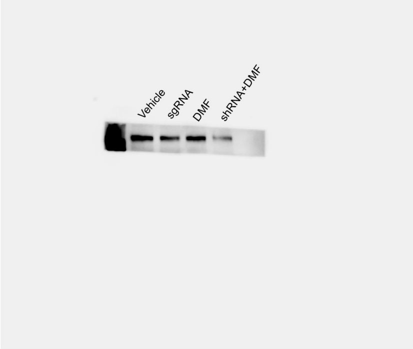

Bax

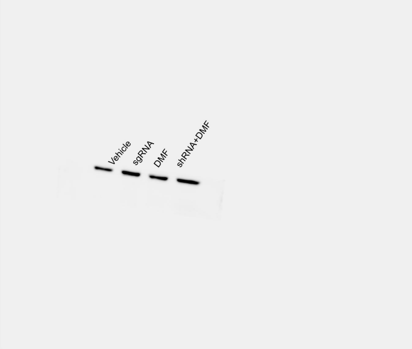

β-actin

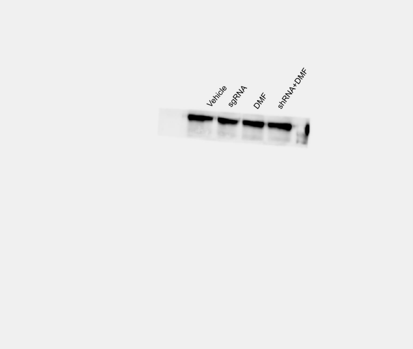

Figure 5F

Bcl-2

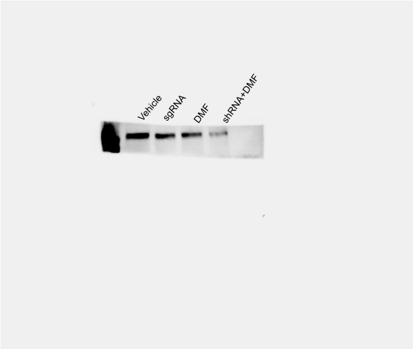

Bax

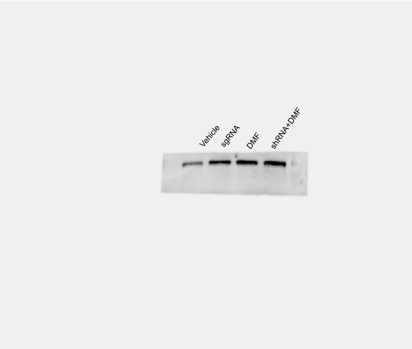

β-actin

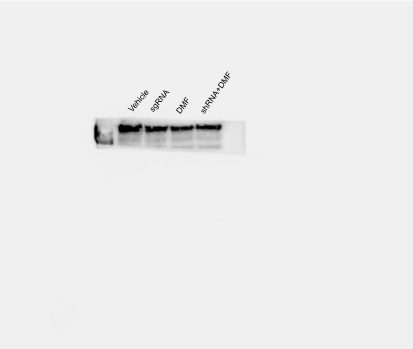

Figure 6E

Bcl-2

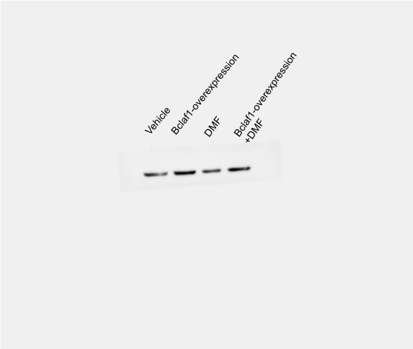

Bax

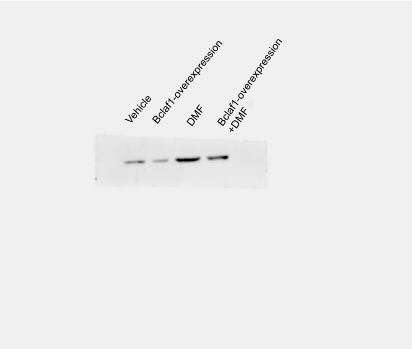

β-actin

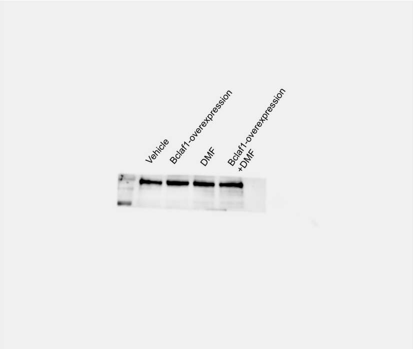

Figure 6F

Bcl-2 Bax β-actin

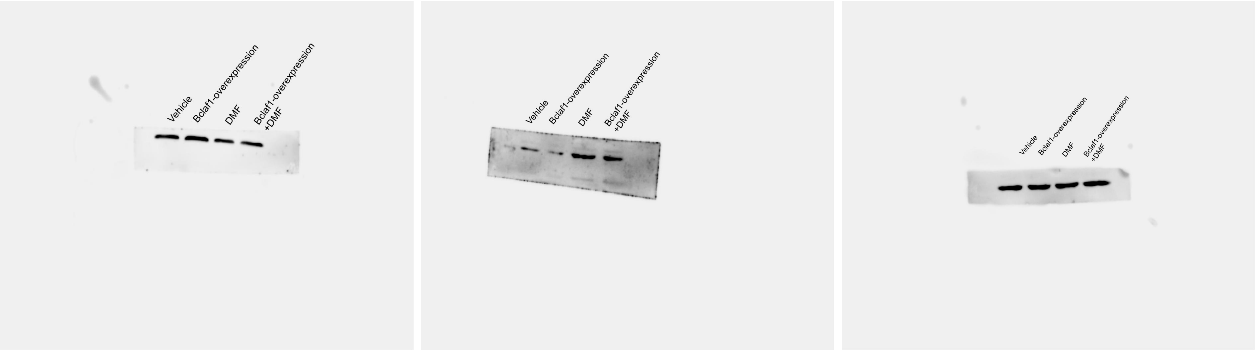

Figure 7E

Bclaf1 β-actin

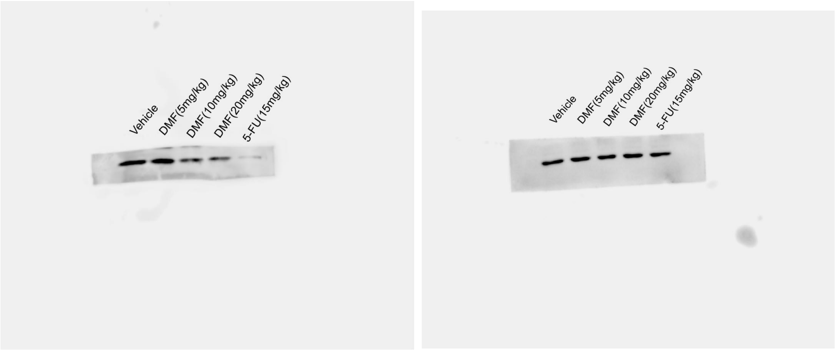

Figure 7F

Bcl-2 Bax β-actin

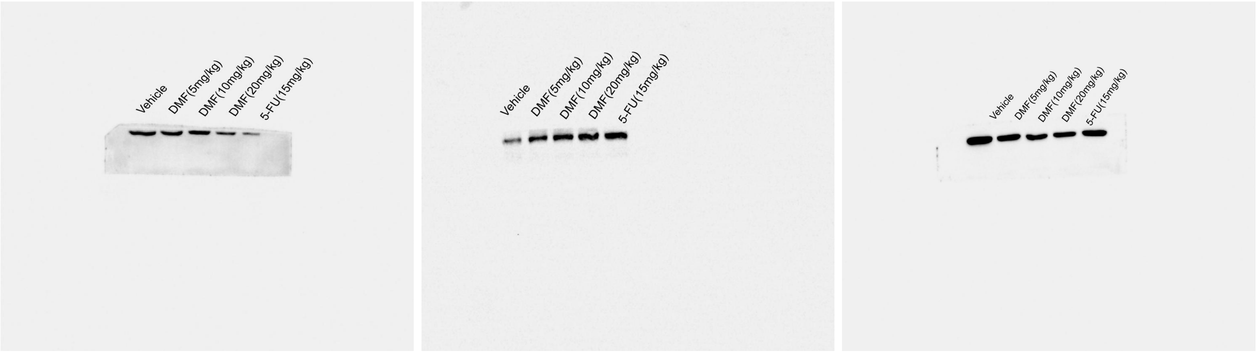

Supplement: Supplementary file 1 [file DataSheet1.PDF]
